# The Common Variant rs11646213 Is Associated with Preeclampsia in Han Chinese Women

**DOI:** 10.1371/journal.pone.0071202

**Published:** 2013-08-19

**Authors:** Ji-peng Wan, Han Zhao, Tao Li, Chang-zhong Li, Xie-tong Wang, Zi-Jiang Chen

**Affiliations:** 1 Department of Obstetrics and Gynecology, Provincial Hospital Affiliated to Shandong University, Jinan, China; 2 Center for Reproductive Medicine, Provincial Hospital Affiliated to Shandong University, Jinan, China; 3 National Research Center for Assisted Reproductive Technology and Reproductive Genetics, Jinan, China; 4 The Key Laboratory for Reproductive Endocrinology of Ministry of Education, Jinan, China; 5 Shandong Provincial Key Laboratory of Reproductive Medicine, Jinan, China; 6 Center for Reproductive Medicine, Renji Hospital, Shanghai Jiao Tong University School of Medicine, Shanghai, China; 7 Shanghai Key Laboratory for Assisted Reproduction and Reproductive Genetics, Shanghai, China; Chinese Academy of Sciences, China

## Abstract

**Background:**

Preeclampsia, characterized by hypertension and proteinuria, is a multifactorial disease caused by complex interactions between environmental and genetic factors. A recent genome-wide association study of blood pressure reported an association between hypertension and rs11646213. This study evaluated the association between preeclampsia and rs11646213.

**Methods:**

A total of 454 cases and 460 controls were recruited to participate in this study. The single nucleotide polymorphism (SNP) rs11646213 was genotyped by polymerase chain reaction (PCR) and direct sequencing.

**Results:**

The allele frequency of rs11646213 was significantly different between the preeclampsia and control groups (P = 0.017, OR = 1.36, 95% CI = 1.06–1.76). Differences were particularly significant in the severe preeclampsia subgroup (P = 0.002, OR = 1.54, 95% CI = 1.17–2.03) and the early-onset preeclampsia subgroup (P = 0.004, OR = 1.57, 95% CI = 1.16–2.13). Genotyping analysis showed that the T allele of rs11646213 could confer a risk for preeclampsia, severe preeclampsia and early-onset preeclampsia.

**Conclusions:**

Rs11646213 upstream of the *CDH13* gene is associated with preeclampsia in Han Chinese women.

## Introduction

Preeclampsia affects approximately 2.5% to 3% of all pregnancies and is a leading cause of maternal morbidity and mortality in developing countries [1]. Although the exact pathogenesis of preeclampsia remains unclear, proposed mechanisms include endothelial cell dysfunction, intravascular inflammation, oxidative stress and angiogenesis imbalance [2]–[4]. Data has shown that daughters and sisters of women with preeclampsia are prone to developing preeclampsia, indicating that genetic factors play a significant role in the pathogenesis [5]. Twin studies estimated the heritability of preeclampsia to be approximately 22%–54% [6], [7].

Epidemiological studies have shown that women with chronic hypertension are at an increased risk of developing preeclampsia and that a family history of chronic hypertension is more common in preeclamptic patients compared with controls [8]–[11]. A genome-wide association study (GWAS) of blood pressure has reported that the single nucleotide polymorphism (SNP) rs11646213 located upstream of the cadherin 13 preprotein (*CDH13*) gene is associated with hypertension in Europeans [12]. Another European study confirmed that the T allele of the SNP rs11646213 conferred a risk for developing hypertension [13].

Thus, we proposed that SNP rs11646213 might be a genetic risk factor for preeclampsia and investigated the association between preeclampsia and rs11646213 in a cohort of Han Chinese women.

## Materials and Methods

### Ethics Statement

The study was approved by the Institutional Review Board of the Provincial Hospital Affiliated to Shandong University. And written informed consent was obtained from each participant.

### Subjects

A total of 914 Han Chinese pregnant women who visited Provincial Hospital Affiliated to Shandong University were enrolled from December 2009 to November 2011. Controls were normotensive women with no antenatal medical or obstetric complications, presenting normal fetal growth at ≥37weeks of gestation (birth weight between the 10th and the 90th percentile), and with a regular postpartum course. Preeclampsia was defined as gestational hypertension with proteinuria (the presence of blood pressure values ≥140/90 mm Hg on 2 measurements at least 6 hours apart; 24 hour urinary protein ≥300 mg or urine dipstick protein≥+) after the 20th week of pregnancy in a previously normotensive and non-proteinuric woman [14]. Blood pressure and proteinuria cut-offs for classifying the cases as mild preeclampsia or severe preeclampsia were greater than 160/110 mmHg and 24 hour urinary protein ≥5 g or urine dipstick protein≥3+, respectively [14]. Early-onset preeclampsia was defined as manifestation before 34 gestational weeks and late-onset preeclampsia thereafter.

Blood pressure was measured in the right arm after a 5-minute period of rest. The following clinical data were collected: maternal age, parity, gestational age at birth, fetal weight and prepregnancy body mass index (pre-BMI). Participants were excluded for the following reasons: a previous renal, autoimmune, metabolic or cardiovascular disease, chronic hypertension, lost to follow-up and multiple pregnancies. Women with gestational hypertension, which is defined as elevated blood pressure without proteinuria, were also excluded from the study.

### Genotyping methods

Genomic DNA was extracted by QIAamp DNA mini kit (Qiagen, Hilden, Germany). Polymerase chain reaction (PCR) conditions were as followings: 95°C for 10 minutes, followed by 34 cycles at 95°C for 30 seconds, 58°C for 30 seconds, 72°C for 45 seconds, and a final step at 72°C for 10 minutes.

The primer sequences were:

Forward: 5′-AAGGAGGGAAGTGTTGACC-3′;

Reverse: 5′-GTCTGGCTTTCATTTCTGG -3′.

The products were analyzed by agarose gel electrophoresis and then were sequenced by an automated sequencer (ABI PRISM 310; Applied Biosystems, Foster City, CA, USA).

### Statistical analysis

General characteristics of the cases and controls were expressed as mean±SD. Linkage disequilibrium (LD) was assessed using Haploview software (Broad Institute, Cambridge, MA, USA). The chi-square test was performed to compare allele frequency. Genetic models were divided into additive (+/+ vs. +/− vs. −/−), dominant (+/+ plus +/− vs. −/−) and recessive (+/+ vs. +/− plus −/−) groups and analyzed by one-way ANOVA. Unconditional logistic regression analysis was used for parity, pre-BMI and maternal age adjustments using SPSS statistical software (version 17.0; SPSS Inc., Chicago, IL, USA). P<0.05 was considered statistically significant.

## Results

The clinical characteristics of the cases and controls are shown in Table 1. Women with preeclampsia had a higher pre-BMI and mean maternal age than the controls (P<0.05). Gestational age at birth and fetal weight were significantly lower among cases compared with controls (P<0.05).

**Table 1:** Clinical characteristics of women with and without preeclampsia.

Characteristics	Control (N = 460)	PE (N = 454)	PE		PE	
			M PE (N = 149)	S PE (N = 305)	Early-onset (N = 211)	Late-onset (N = 243)
Maternal age	28.1±5.0	29.0±5.5^a^	28.6±5.3	29.2±5.6^a^	29.4±5.3^a^	28.7±5.7
Pre-BMI	22.1±3.2	24.2±3.9^a^	23.4±3.5^a^	24.5±3.9^a^^,^ b	24.9±3.9^a^	23.5±3.7^a^^,^ c
Primiparas	264(57.4%)	280 (61.7%)	91(61.1%)	189(61.9%)	123(58.3%)	157(64.6%)
SBP(mmHg)	118.6±10.0	163.1±20.1^a^	148.4±15.1^a^	170.2±18.3^a^^,^ b	169.3±20.4^a^	157.8±18.3^a^^,^ c
DBP(mmHg)	76.3±7.1	107.2±14.2^a^	99.6±12.1^a^	114.8±13.4^a^^,^ b	115.2±14.1^a^	105.3±13.9^a^^,^ c
Delivery weeks	39.2±1.3	35.9±3.5^a^	36.8±2.9^a^	35.1±3.7^a^^,^ b	33.1±3.3^a^	37.5±2.5^a^^,^ c
Fetal weight(g)	3398.5±482.8	2539.7±926.9^a^	2818.1±970.1^a^	2371.2±875.1^a^^,^ b	1951.4±746.9^a^	2966.1±797.1^a^^,^ c

Notes: PE = preeclampsia; MPE = mild preeclampsia; SPE = severe preeclampsia;

Pre –BMI = prepregnancy body mass index; SBP = Blood pressure, systolic; DBP = Blood pressure, diastolic.

a P value<0.05 VS control.

b P value<0.05 VS M PE.

c P value<0.05 VS early-onset PE.

No deviation of allele frequencies from Hardy–Weinberg equilibrium was found in both the cases and controls. As shown in Table 2, the minor allele frequency (MAF) was significantly different between the case and control groups (P = 0.017, OR = 1.36, 95% CI = 1.06–1.76). Relative to the controls, an association with rs11646213 was observed in women with severe (P = 0.002, OR = 1.54, 95% CI = 1.17–2.03), but not mild preeclampsia (P = 0.91, OR = 1.02, 95% CI = 0.70–1.50). The MAF was also significantly different between early-onset preeclampsia and the controls (P = 0.004, OR = 1.57, 95% CI = 1.16–2.13), although no difference was found between late-onset preeclampsia and controls (P = 0.27, OR = 1.19, 95% CI = 0.87–1.62).

**Table 2:** Allele frequencies of rs11646213 in women with and without preeclampsia.

		Allele (T/A)	MAF	P	OR(95%CI)	P_adjust_ a
**Control**		124/796	0.135			
**PE**		159/749	0.175	**0.017**	**1.36(1.06–1.76)**	**0.046**
**PE**	**M PE**	41/257	0.138	0.91	1.02(0.70–1.50)	0.352
	**S PE**	118/492	0.193	**0.002**	**1.54(1.17–2.03)**	0.054
**PE**	**Early-onset**	83/339	0.197	**0.004**	**1.57(1.16–2.13)**	**0.040**
	**Late-onset**	76/410	0.156	0.27	1.19(0.87–1.62)	0.330

Notes: PE = preeclampsia; MAF = minor allele frequency; OR = odds radio between case and control group; 95%CI = 95% confidence interval.

a The P value was adjusted by parity, pre-BMI and maternal age.

Women with preeclampsia were further divided into four subgroups: early-onset mild preeclampsia (28 cases), early-onset severe preeclampsia (183 cases), late-onset mild preeclampsia (121 cases) and late-onset severe preeclampsia (122cases). The MAF was significantly different between early-onset severe preeclampsia and controls (P = 0.003). No significant differences were observed between the controls and other subgroups (Table S1).

Genotype frequency was further analyzed under additive, recessive and dominant models (Table 3). Significant differences were identified between cases and controls under the additive (P = 0.040) and dominant models (P = 0.010). There was a significant difference between severe preeclampsia and the controls under additive (P = 0.006) and dominant models (P = 0.001). Similar results were observed in the early-onset group under additive (P = 0.013) and dominant models (P = 0.019).

**Table 3:** Genotype frequencies of rs11646213 in women with and without preeclampsia.

		Frequency (AA/AT/TT)	P_add_	P_dom_	P_rec_
**Control**		344/108/8			
**PE**		305/139/10	**0.040**	**0.010**	0.610
**PE**	**MPE**	110/37/2	0.901	0.820	0.740
	**SPE**	195/102/8	**0.006**	**0.001**	0.400
**PE**	**Early-onset**	137/65/9	**0.013**	**0.009**	0.053
	**Late-onset**	168/74/1	0.053	0.110	0.140

Notes: PE = preeclampsia; MPE = mild preeclampsia; SPE = severe preeclampsia;

P_add_: P value of additive model (three genotypes).

P_dom_: P value of dominant model [(homozygotes of risk allele+heterozygotes) vs. homozygotes of non-risk allele].

P_rec_: P value of recessive model [homozygotes of risk allele vs. (heterozygotes+homozygotes of non-risk allele)].

The T allele is the risk allele.

## Discussion

In the present study, a significant association between preeclampsia and rs11646213 was identified in a cohort of Han Chinese women. When the cases were divided into subgroups, there was a significant association between early-onset severe preeclampsia and the controls.

The participants were all Han Chinese women in our study, so that there was no variation in genetic background. The T allele, which confers an increased risk of developing preeclampsia, was the minor allele. As shown in the GWAS blood pressure study, the A allele was identified as the minor allele in a European population [12], demonstrating racial variations of rs11646213.

*CDH13*, located on chromosome 16q24, encodes cadherin-13 and is highly expressed in the cardiovascular system [15]. Cadherin-13 plays an important role in many biological processes, including vascular wall remodeling, modulating angiogenesis and protecting endothelial cells from oxidative stress-induced apoptosis [16], [17], most of which are involved in the pathogenesis of preeclampsia. Cadherin-13 has been identified as the receptor for adiponectin in vascular endothelial and smooth muscle cells [18], [19]. Several GWA studies have reported a significant association between plasma adiponectin levels and genetic variants of *CDH13* [20]–[24]. Based on the close association between adiponectin and preeclampsia [25]–[29], it is rational to speculate that *CDH13* may be involved in the pathogenesis of preeclampsia through interactions with adiponectin.

Preeclampsia is currently recognized as a syndrome rather than a definite disease. In this study, significant differences in genotype distribution were observed between early-onset severe preeclampsia and the controls after the cases were divided into subgroups. These results are in agreement with the theory that defective placental angiogenesis plays an important role in the development of early-onset preeclampsia, while late-onset preeclampsia generally has a normal placenta morphology [30], [31]. Severe preeclampsia, which usually develops earlier than mild preeclampsia, has been closely associated with impaired placentation [31].

Several limitations should be considered. First, genetic factors that influence the pathogenesis of preeclampsia differ by ethnic group, and the association between *CDH13* and preeclampsia need to be confirmed in a larger sample size composed of different ethnic groups. Second, the functional significance of the SNP rs11646213 remains unknown and molecular mechanisms regarding *CDH13* in the pathophysiology of preeclampsia should be examined in future studies. Third, while we limited our study to only one genetic variant of *CDH13*, future studies should evaluate associations between preeclampsia and other genetic variants, particularly those that lack strong linkage disequilibrium with rs11646213.

In conclusion, we identified a significant association between rs11646213 and preeclampsia in Han Chinese women.

## Supporting Information

Table S1 **Allele frequencies of rs11646213 in control, early-onset mild preeclampsia, early-onset severe preeclampsia, late-onset mild preeclampsia and late-onset severe preeclampsia subjects.** PE: preeclampsia; MAF: minor allele frequency; OR: odds radio; 95% CI: 95% confidence interval.
(DOCX)
