# Supplementary material for: The Common Variant rs11646213 Is Associated with Preeclampsia in Han Chinese Women
Source: PLoS One. 2013 Aug 19;8(8):e71202. doi: 10.1371/journal.pone.0071202 (PMC3747203; doi:10.1371/journal.pone.0071202)
Supplement: Table S1 — Allele frequencies of rs11646213 in control, early-onset mild preeclampsia, early-onset severe preeclampsia, late-onset mild preeclampsia and late-onset severe preeclampsia subjects. PE: preeclampsia; MAF: minor allele frequency; OR: odds radio; 95% CI: 95% confidence interval. (DOCX) [file pone.0071202.s001.docx]

Table S1 Allele frequencies of rs11646213 in control, early-onset mild preeclampsia, early-onset severe preeclampsia, late-onset mild preeclampsia and late-onset severe preeclampsia subjects.

|  | **Allele（T/A）** | **MAF** | **P** | **OR(95%CI)** | **P_adjust_^a^** |
| --- | --- | --- | --- | --- | --- |
| **Control** | 124/796 | 0.135 |  |  |  |
| **Early-onset mild PE** | 9/47 | 0.161 | 0.583 | 1.23(0.59-2.57) | 0.351 |
| **Early-onset severe PE** | 74/292 | 0.202 | **0.003** | 1.63(1.18-2.24) | **0.043** |
| **Late-onset mild PE** | 32/210 | 0.132 | 0.918 | 0.98(0.64-1.49) | 0.522 |
| **Late-onset severe PE** | 44/200 | 0.180 | 0.072 | 1.41(0.97-2.06) | 0.494 |

Notes: PE =preeclampsia; MAF = minor allele frequency; OR = odds radio between case and control group; 95%CI= 95% confidence interval.

^a^ The P value was adjusted by parity, pre-BMI and maternal age.
